# National Early Warning Score 2 (NEWS2) and 3-level triage scale as risk predictors in frail older adults in the emergency department

**DOI:** 10.1186/s12873-020-00379-y

**Published:** 2020-10-28

**Authors:** Kirsi Kemp, Janne Alakare, Veli-Pekka Harjola, Timo Strandberg, Jukka Tolonen, Lasse Lehtonen, Maaret Castrén

**Affiliations:** 1 Emergency Medicine, Helsinki University, Helsinki, Finland; 2 Department of Emergency Medicine and Services, Helsinki University Hospital, Helsinki, Finland; 3 University of Helsinki, Clinicum, and Helsinki University Hospital, Helsinki, Finland; 4 University of Oulu, Center for Life Course Health Research, Oulu, Finland; 5 Department of Public Health, University of Helsinki and Helsinki University Hospital, Helsinki, Finland

**Keywords:** Emergency department, Triage, Frailty, Older adults

## Abstract

**Background:**

The aim of the emergency department (ED) triage is to recognize critically ill patients and to allocate resources. No strong evidence for accuracy of the current triage instruments, especially for the older adults, exists. We evaluated the National Early Warning Score 2 (NEWS2) and a 3-level triage assessment as risk predictors for frail older adults visiting the ED.

**Methods:**

This prospective, observational study was performed in a Finnish ED. The data were collected in a six-month period and included were ≥ 75-year-old residents with Clinical Frailty Scale score of at least four. We analyzed the predictive values of NEWS2 and the three-level triage scale for 30-day mortality, hospital admission, high dependency unit (HDU) and intensive care unit (ICU) admissions, a count of 72-h and 30-day revisits, and ED length-of-stay (LOS).

**Results:**

A total of 1711 ED visits were included. Median for age, CFS, LOS and NEWS2 were 85 years, 6 points, 6.2 h and 1 point, respectively. 30-day mortality was 96/1711. At triage, 69, 356 and 1278 of patients were assessed as red, yellow and green, respectively. There were 1103 admissions, of them 31 to an HDU facility, none to ICU.

With NEWS2 and triage score, AUCs for 30-day mortality prediction were 0.70 (0.64–0.76) and 0.62 (0.56–0.68); for hospital admission prediction 0.62 (0.60–0.65) and 0.55 (0.52–0.56), and for HDU admission 0.72 (0.61–0.83) and 0.80 (0.70–0.90), respectively.

The NEWS2 divided into risk groups of low, medium and high did not predict the ED LOS (*p* = 0.095). There was a difference in ED LOS between the red/yellow and as red/green patient groups (*p* < 0.001) but not between the yellow/green groups (*p* = 0.59).

There were 48 and 351 revisits within 72 h and 30 days, respectively. With NEWS2 AUCs for 72-h and 30-day revisit prediction were 0.48 (95% CI 0.40–0.56) and 0.47 (0.44–0.51), respectively; with triage score 0.48 (0.40–0.56) and 0.49 (0.46–0.52), respectively.

**Conclusions:**

The NEWS2 and a local 3-level triage scale are statistically significant, but poor in accuracy, in predicting 30-day mortality, and HDU admission but not ED LOS or revisit rates for frail older adults. NEWS2 also seems to predict hospital admission.

## Background

Frailty as a geriatric syndrome has been associated with increased morbidity and mortality in the emergency department [1]. With ageing population and crowded emergency departments (ED), robust tools are needed for identifying older adults with critical or high-risk conditions. However, evidence regarding the reliability of ED screening instruments for frail older adults is limited.

ED triage for screening patients in high risk is utilized at the time of ED admission. In recent years, early warning scores have become widely used as screening tools, not only for detecting deterioration in follow-up on hospital wards, but for initial assessment, too. Both tools are based on vital signs, which are less reliable in the older adult population due to chronic illness and polypharmacy [2].

There is no strong evidence for any of the current triage instruments, although five-level instruments seem to be more accurate than three-level instruments [3]. Triage seems to be less reliable for older adults: the emergency severity index, seemed to recognize less than half of older adults requiring a life-saving procedure [4]. According to LaMantia et al., sensitivity and specificity of an abnormal vital sign taken at triage for predicting death or admission to an intensive care unit (ICU) were 73 and 50% respectively [2]. Patients who are undertriaged to a less urgent group may have increased morbidity and mortality due to longer waiting times and longer emergency department length-of-stay (LOS).

The evidence for early warning scores at the emergency department so far is limited: studies mostly include small or pre-selected sub-cohorts. One recent study showed that the national early warning score (NEWS2) is independently associated with mortality and ICU admissions [5]. Another study found that the modified early warning score (MEWS) adequately predicted hospitalization and in-hospital mortality for the older adults in the emergency department [6].

In the past, other tools such as the Identification of Seniors at Risk (ISAR) and the Triage Risk Stratification Tool (TRST) have been used for screening older adults in the emergency department. Neither of these instruments performed well enough to be used as the sole screening tool [7, 8]. The Canadian ED frailty index tool seemed to predict adverse outcomes in individual studies [9, 10].

Vital sign measuring with or without NEWS2-scoring at the time of admission, and scaled triage methods, are used in most ED’s. These tools may be useful for early recognition of critically ill patients among the frail older adults and may help improving outcomes with early treatment. However, it is essential to understand the possible limitations in predictive accuracy of these methods. In this prognostic study we evaluate the predictive value of the NEWS2 and a three-level ED triage scale for mortality, hospital admission, high dependency unit (HDU) or ICU admission, LOS in the ED and ED-readmissions, in 75 year or older patients with frailty.

## Methods

**Fig. 1:**
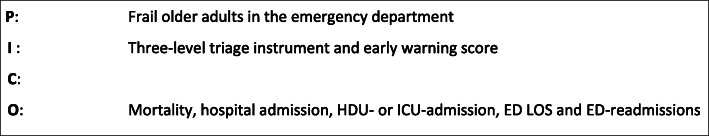
PICO statement for ED outcome prediction for the frail older adults

The study is a prospective, observational cohort study. It is registered with primary and secondary outcome measures in context of eligibility screening and patient enrollment for the GAOPS-trial (Clinicaltrials.gov registration NCT03783234). Prior to collecting data, the study was supported by a statement from the ethical board of University of Helsinki and Helsinki University Hospital (HUS/1171/2018). A permission for the study was granted by Helsinki University Hospital (HUS/278/2018). The PICO statement is presented in Fig. 1.

### Data collection

The study was run in Helsinki University Hospital Emergency Department in Espoo, which is a medium sized emergency department with about 60,000 adult patient visits per annum. The emergency department utilizes a local three-level triage instrument with levels red (emergent) yellow (urgent) and green (standard) (Appendix 1). For assessing frailty, we used the Clinical Frailty Scale (CFS) by Rockwood et al. [11].

Inclusion criteria for the patients visiting the ED during the study period were the following: 1) registration as resident in the hospital district 2) age of 75 or more at the time of the ED visit 3) nurse-assessed CFS score of four or higher at the time of the ED visit.

Pre-specified primary outcome measure was mortality after the ED admission during the 30-day follow up. Pre-specified secondary outcome measures were: 1) hospital admission from the ED 2) HDU/ICU admission from the ED 3) readmission to the ED in 72 h and 30 days. ED LOS was added to the analysis for secondary outcome.

Data were collected prospectively in a 6-month period between December 11, 2018 and June 7, 2019. All visits of eligible patients (age ≥ 75 at the time of the ED visit, registered as resident of the municipalities in the district of the hospital) were given an individual code by the secretary at ED admittance process.

A coded paper form was delivered to the nurse treating the eligible patient. ED nurses filled the forms in two steps: First the CFS was assessed. Then, if the CFS was at least 4, nurses were guided to record the NEWS2, patient’s co-morbidities and social background. Additional data regarding triage class of the ED visit, admission or discharge information, LOS in the ED, 30-day follow-up of mortality status with time of death were collected from electronic health record (EHR) by the researchers.

### Analysis

Data were analyzed with the SPSS program using the AUROC test for parametric data and ANOVA for continuous data. We analyzed the data by NEWS2 and triage category testing differences in 30- day mortality, ED LOS, hospital and ICU/HDU admission. For the clarity of presentation of the results for ED LOS, the NEWS2 were grouped into low (0–4), moderate [5, 6] and high (> = 7), in line with the Royal College of Physicians guideline [12].

## Results

**Fig. 2:**
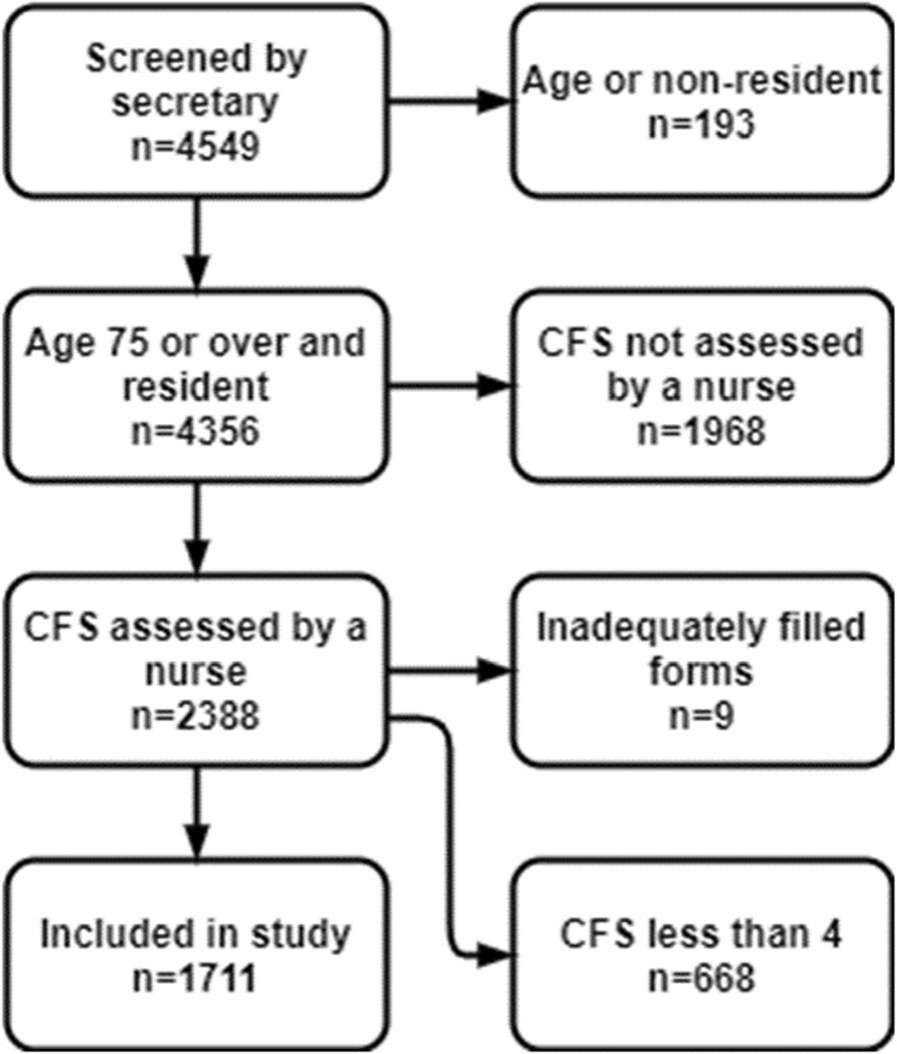
Patient selection flowchart

A total of 4549 patient visits were screened. After excluding patients not meeting the first two criteria (aged less than 75 at the time of visit or registered as resident of municipalities in other hospital districts, *n* = 193) a total of 4356 patient visit codes were registered. For eligible patient visits a total of 2388 forms were returned filled with the nurse assessed CFS (55% of the forms). Nine forms were incorrectly filled and were excluded, leaving us with 2379 visits for analysis. Of the correctly filled forms, there were 1711/2379 visits with a CFS score of at least four (72%), and 668/2379 (28%) with CFS score of less than four. There were 1304 individual patients included in the study, with a total of 412/1711 (24%) revisits (Fig. 1). Follow-up data from electronic health records were available for all included visits (*n* = 1711). Flowchart for patient selection is described in Fig 2.

Of the visits 664 (39%) were male patients and 1047 (61%) were female patients. Mean and median age was 85 years. Median CFS was 6. Mean ED LOS was 8.6 and median ED LOS was 6.2 h. Median NEWS2-score was 1. There were a total of 412/1711 revisits during the 6-month study period. Of these revisits 351/1711 (20.5%) within 30 days and 48/1711 (2.8%) within 3 days. 96/1711 patients deceased within 30 days from their visit. 69/1711 (4.0%) patient visits were triaged as red and 356/1711 (20.8%) were triaged as yellow, data were missing for 8 visits. The remaining 1278/1711 (74.7%) were triaged as green.

The hospital admission rate was 64.4% (1103/1711) patients were admitted. Of those, 31 were admitted to an HDU facility, yet there were no ICU admissions.

### Mortality

**Fig. 3:**
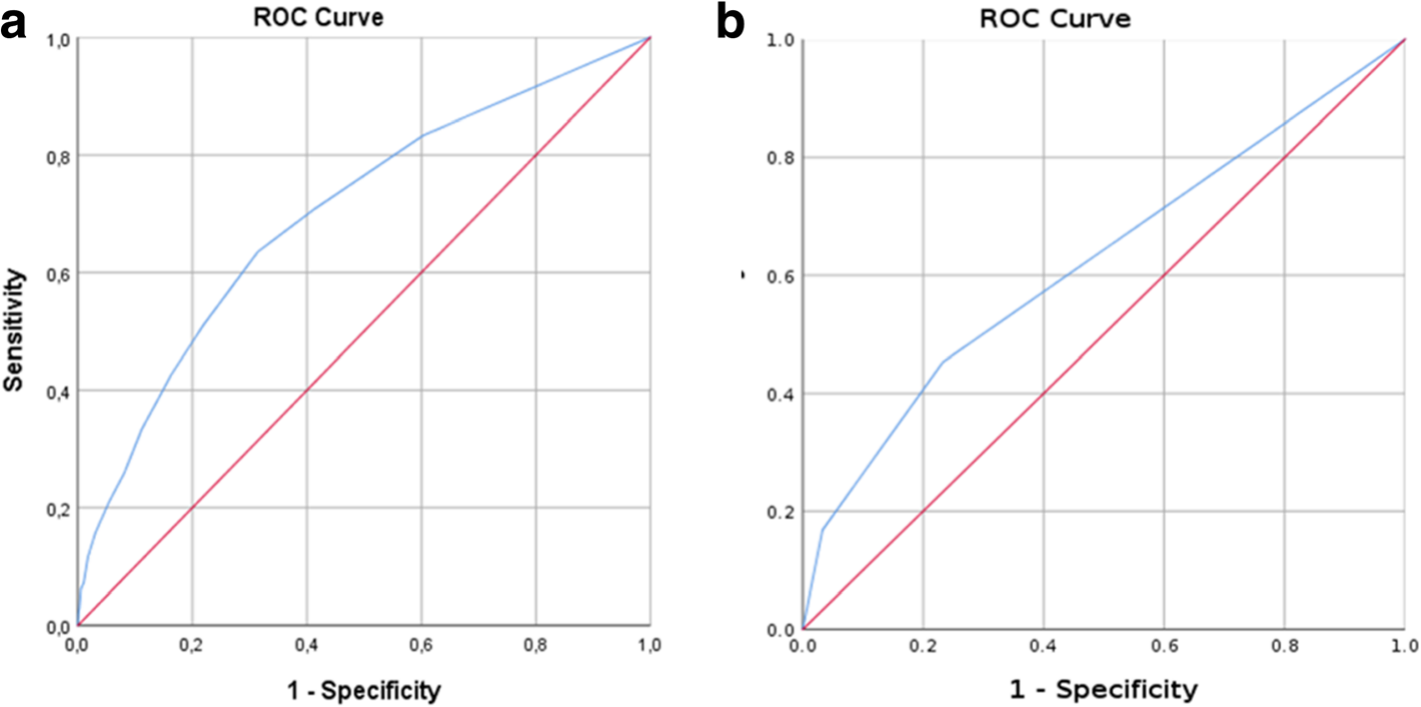
Mortality prediction with the NEWS2 score (left) and the 3-level triage instrument (right)

Patients with higher NEWS2 score had significantly increased 30-day mortality (*p* < 0.001). In the ROC analysis AUC was 0.70 (95% CI 0.64–0.76) (Fig. 3a).

Mortality differed significantly between triage groups (*p* < 0.001). In the red group, mortality was 23.2% (16/69); in the yellow group, 7.6% (27/356); and in the green group, 4.1% (52/1278). In the ROC analysis AUC was 0.62 (95% CI 0.56–0.68) (Fig. 3b).

### Hospital admission

Patients with higher NEWS2 scores were more frequently admitted (*p* < 0.001). 42/43 (97.7%) patients with a NEWS2 score of at least 8 were admitted. 238/296 (80.4%) of patients with a NEWS2 score between 4 and 7 were admitted. Of those with a NEWS2 score of 3 or lower, 762/1308 (58.3%) were admitted. In the ROC analysis, AUC was 0.62 (95% CI 0.60–0.65).

There was a difference in hospital admission rates between triage groups (*p* < 0.001). For patients in the red triage group admission rate was 94.2% (65/69); for the yellow group, admission rate was 68.5% (244/356); and for the green group 61.8% (790/1278). The AUC was 0.55 (95% CI 0.52–0.56) in the ROC analysis.

### HDU admission

**Fig. 4:**
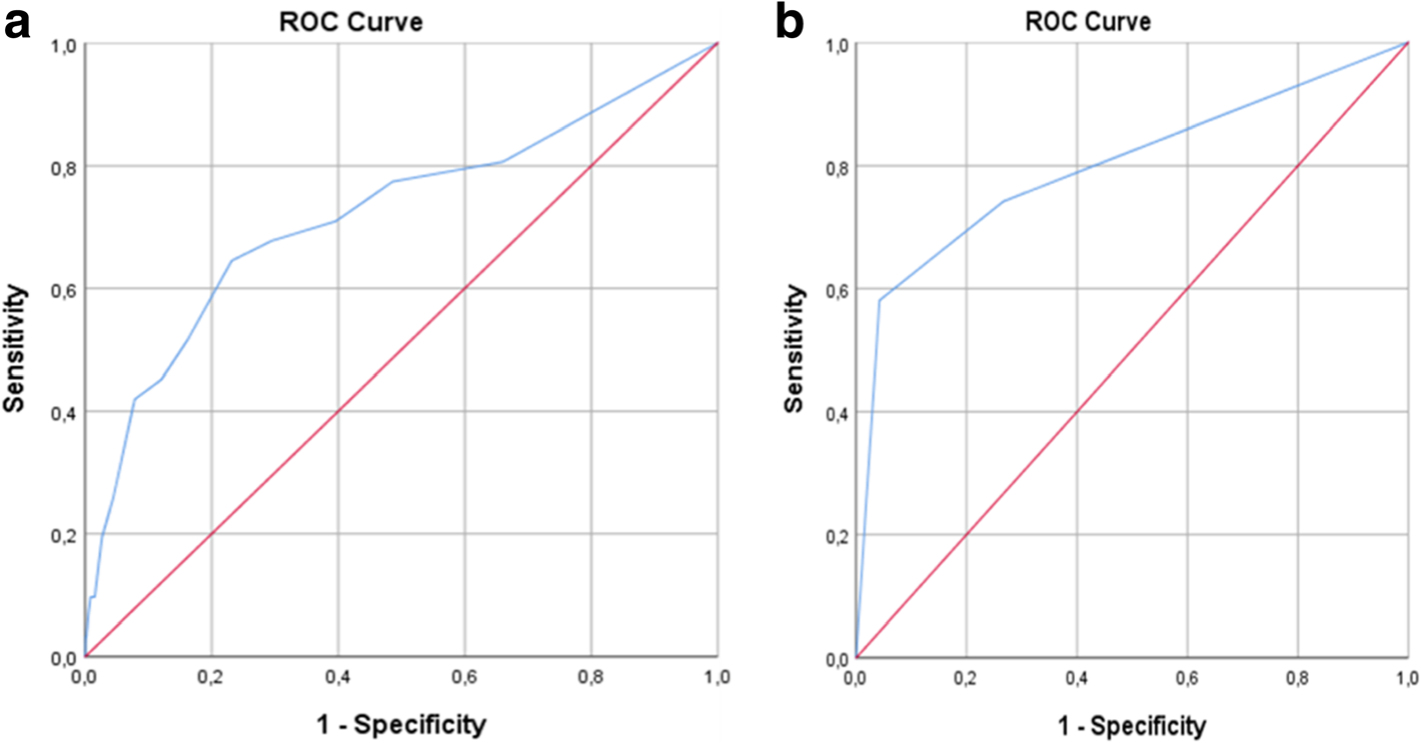
HDU admission prediction with the NEWS2 score (left) and a 3-level triage instrument (right)

Of the 1102 admitted patients, 31 (2.8%) were admitted to an HDU facility. There were no ICU admissions from the ED in this study population. There was a significant increase in HDU admissions for patients with higher NEWS2 scores (*p* < 0.001). The ROC analysis shows an AUC value of 0.72 (95% CI 0.61–0.83) (Fig. 4a).

There was a significant increase in HDU admissions in the red and yellow triage categories (*p* < 0.001). In the red group, 18/63 (28.6%) patients were admitted to HDU. In the yellow and green groups, the numbers of HDU admissions were 5/243 (2.1%) and 8/786 (1.0%), respectively. The ROC analysis shows AUC value of 0.80 (95% CI 0.70–0.90) (Fig. 4b).

### Ed los

Mean LOS for the red group was 4.8 h (95% CI 4.2–5.5), for the yellow group 8.45 h (95% CI 7.8–9.1) and 8.8 h for the green group (95% CI 8.46–9.2). There was a significant difference in ED LOS between the red and the yellow patients (*p* < 0.001) but not between the yellow and the green groups (*p* = 0.59) (Table 1).

**Table 1:** Predictive values of triage score and NEWS2 for ED LOS

		Mean Difference (h)	Std. Error	Sig.	95% CI	
					Lower	Upper
**Triage group**						
Red	Yellow	−3.64^*^	0.852	< 0.001	−5.63	−1.64
Red	Green	−4.02^*^	0.800	< 0.001	−5.90	−2.14
Yellow	Green	−0.380	0.388	< 0.001	−1.29	0.53
**News2 risk group**						
Low	Moderate	0.07	0.564	0.993	−1.26	1.39
Low	High	1.19	0.549	0.077	−0.10	2.48
Moderate	High	1.13	0.748	0.289	−0.63	2.88

There were 1406 patient visits with a low NEWS2 score, mean LOS for this group was 8.67 h (95% CI 8.33–9.02). There were 148 patients with a moderate NEWS2 score, mean LOS of 8.61 h (95%CI 7.61–9.60) and 157 patients with a high NEWS2 score with a mean LOS of 7.48 h (95% CI 6.58–8.39). There were no significant differences between the groups (*p* = 0.095) the exact figures are presented in Table 1.

### Revisitation

There were 351 revisits within 30 days and 48 revisits within 3 days of the index visit. For all visits the AUC for 30- and 3-day revisit prediction with the NEWS2 score are 0.47 (95% CI 0.44–0.51) (*p* = 0.13) and 0.48 (95% CI 0.40–0.56) (*p* = 0.61) respectively. The AUC for 30- and 3-day revisit prediction with the triage score are 0.49 (95% CI 0.46–0.52) (*p* = 0.57) and 0.48 (95% CI 0.40–0.56) (*p* = 0.63) respectively.

In a post-hoc analysis for non-admitted patients, NEWS2 score did not predict 3-day revisitation (*p* = 0.77, AUC 0.52 (95% CI 0.41–0.62)), nor did triage score (*p* = 0.89, AUC 0.51 (95%CI 0.41–0.61)).

## Strengths and limitations

The strength of our study is that we were able to include a relatively large study population. We had access to thorough documentation in the electronic patient records. We have systematically attempted to reduce bias by completing the STROBE checklist for cohort studies to assess bias (Appendix 2).

Our study was completed in a single centre, which might contribute to selection bias. The three-level triage tool that was used, has not been formally validated, thus our results might not be applicable to other ED’s that utilize different triage instruments. However, three-level triage instruments have been shown to be less sensitive compared to five-level instruments; therefore, any findings on a three-level instruments could be argued to be significant.

CFS was not assessed for almost a half of potentially eligible patients. Our hypothesis is that this happened especially due to crowding. This might contribute to selection bias, but the selection of patients was done independently from the researchers, which in turn might be a redeeming factor.

## Discussion

Our analysis shows that the NEWS2 predicts 30-day mortality and HDU-admission with modest accuracy for frail older adult ED patients. The 3-level triage performed on admission to the ED predicts 30-day mortality with relatively low accuracy and HDU admission with modest accuracy on this patient group. NEWS2 also seems to predict hospital admission but the accuracy remains relatively low. Our triage scale or the NEWS2 did not predict revisitation rates for the frail elderly at 3 or 30 days.

These findings are in keeping with previous studies but the accuracy for mortality was lower in our study [6]. This is probably inherent to our inclusion criteria of frailty which is likely to make vital signs less reliable predictor for adverse outcomes [2].

There were no ICU admissions in our study population but 2.8% of the admitted patients were admitted to a HDU. The Finnish HDU’s equal level 2 care, which includes for example noninvasive ventilation or use of vasoactive drugs but not invasive mechanical ventilation. Many of our patients were severely frail and therefore mechanical ventilation may have been decided to be unfeasible for them; many patients had advance care planning against more intensive treatments. We note that both the NEWS2 and the 3-level triage predicted HDU admissions more accurately than 30-day mortality, which suggests that these tools are valuable in recognizing critically ill patients in need of high-level care.

Neither NEWS2 nor our triage instrument were able to predict revisitation rates. This was an expected finding, as we assume that any patient with high a triage level or NEWS2 would have been admitted on their first visit.

We found a difference in ED LOS between patients who were triaged as red and yellow, but not between the other groups. The NEWS2 does not seem to predict ED LOS in our department. The mean LOS was high for all patients except for those who were triaged as red. We hypothesize that this might be due to exit block and crowding in our department. Some of the previous studies suggest that prolonged ED LOS is associated with adverse outcomes, but it has not been shown whether this is an independent risk in the older frail adult population [13].

## Conclusion

NEWS2 and a local 3-level triage are statistically significant in predicting 30-day mortality, and HDU admission but not ED LOS or revisit rates for frail older adults. NEWS2 also seems to predict hospital admission. However, accuracy defined by AUC for mortality and hospital admissions are poor for both predictors. This supports previous findings that more robust risk prediction models are needed for older frail patients visiting EDs.

## Figures and Tables

**Table 2:**
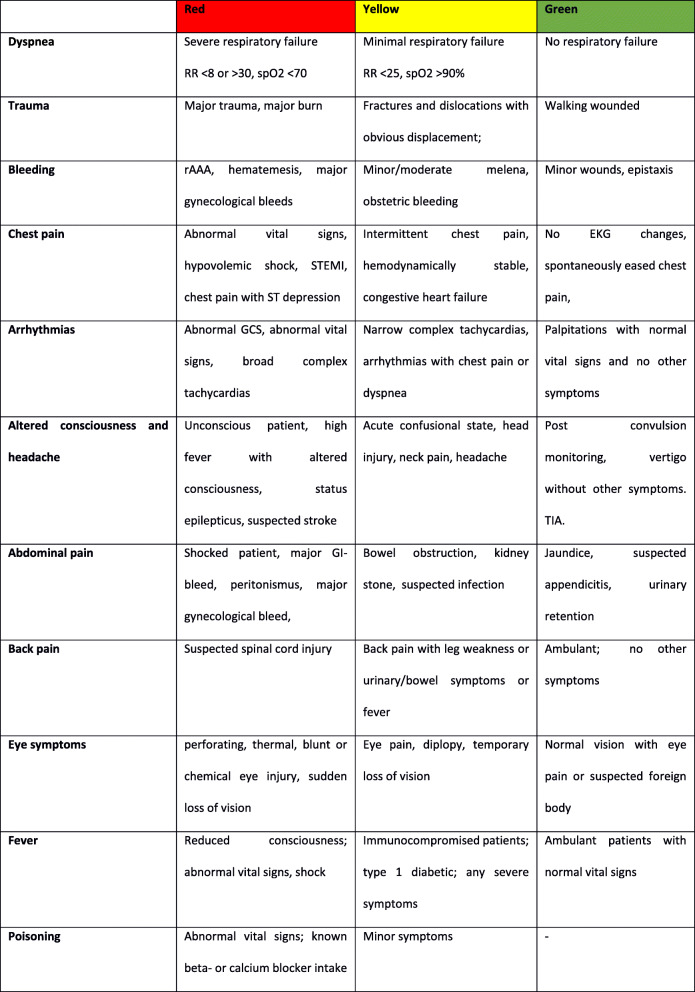
Our 3 level triage tool (abbreviated and translated from original Finnish version)

## Data Availability

The datasets used and/or analysed during the current study are available from the corresponding author on reasonable request.

## Appendix 2

### The STROBE checklist for cohort studies

**Table 3:** STROBE Statement—Checklist of items that should be included in reports of ***cohort studies***

	Item No	Recommendation	Observed
**Title and abstract**	1	(*a*) Indicate the study’s design with a commonly used term in the title or the abstract	Yes
		(*b*) Provide in the abstract an informative and balanced summary of what was done and what was found	Yes
**Introduction**			
Background/ rationale	2	Explain the scientific background and rationale for the investigation being reported	Yes
Objectives	3	State specific objectives, including any prespecified hypotheses	Yes
**Methods**			
Study design	4	Present key elements of study design early in the paper	Yes
Setting	5	Describe the setting, locations, and relevant dates, including periods of recruitment, exposure, follow-up, and data collection	Yes
Participants	6	(*a*) Give the eligibility criteria, and the sources and methods of selection of participants. Describe methods of follow-up	Yes
		(*b*) For matched studies, give matching criteria and number of exposed and unexposed	Yes
Variables	7	Clearly define all outcomes, exposures, predictors, potential confounders, and effect modifiers. Give diagnostic criteria, if applicable	Yes (where applicable)
Data sources/ measurement	8^a^	For each variable of interest, give sources of data and details of methods of assessment (measurement). Describe comparability of assessment methods if there is more than one group	Yes (where applicable)
Bias	9	Describe any efforts to address potential sources of bias	Yes
Study size	10	Explain how the study size was arrived at	Yes
Quantitative variables	11	Explain how quantitative variables were handled in the analyses. If applicable, describe which groupings were chosen and why	Yes
Statistical methods	12	(*a*) Describe all statistical methods, including those used to control for confounding	Yes
		(*b*) Describe any methods used to examine subgroups and interactions	Yes
		(*c*) Explain how missing data were addressed	N/A
		(*d*) If applicable, explain how loss to follow-up was addressed	N/A
		(*e*) Describe any sensitivity analyses	N/A
**Results**			
Participants	13^a^	(a) Report numbers of individuals at each stage of study—eg numbers potentially eligible, examined for eligibility, confirmed eligible, included in the study, completing follow-up, and analysed	Yes
		(b) Give reasons for non-participation at each stage	Yes
		(c) Consider use of a flow diagram	Yes
Descriptive data	14^a^	(a) Give characteristics of study participants (eg demographic, clinical, social) and information on exposures and potential confounders	Yes
		(b) Indicate number of participants with missing data for each variable of interest	N/A
		(c) Summarise follow-up time (eg, average and total amount)	Yes
Outcome data	15^a^	Report numbers of outcome events or summary measures over time	Yes
Main results	16	(*a*) Give unadjusted estimates and, if applicable, confounder-adjusted estimates and their precision (eg, 95% confidence interval). Make clear which confounders were adjusted for and why they were included	Yes
		(*b*) Report category boundaries when continuous variables were categorized	Yes
		(*c*) If relevant, consider translating estimates of relative risk into absolute risk for a meaningful time period	N/A
Other analyses	17	Report other analyses done—eg analyses of subgroups and interactions, and sensitivity analyses	Yes
**Discussion**			
Key results	18	Summarise key results with reference to study objectives	Yes
Limitations	19	Discuss limitations of the study, taking into account sources of potential bias or imprecision. Discuss both direction and magnitude of any potential bias	Yes
Interpretation	20	Give a cautious overall interpretation of results considering objectives, limitations, multiplicity of analyses, results from similar studies, and other relevant evidence	Yes
Generalisability	21	Discuss the generalisability (external validity) of the study results	Yes
**Other information**			
Funding	22	Give the source of funding and the role of the funders for the present study and, if applicable, for the original study on which the present article is based	Yes

^a^Give information separately for exposed and unexposed groups

**Note:** An Explanation and Elaboration article discusses each checklist item and gives methodological background and published examples of transparent reporting. The STROBE checklist is best used in conjunction with this article (freely available on the Web sites of PLoS Medicine at http://www.plosmedicine.org/, Annals of Internal Medicine at http://www.annals.org/, and Epidemiology at http://www.epidem.com/). Information on the STROBE Initiative is available at http://www.strobe-statement.org

## Abbreviations

ANOVA: Analysis of variance
AUC: Area under the curve
AUROC: Area under receiver operating characteristic
CFS: Clinical frailty scale
ED: Emergency department
EHR: Electronic health record
FI-ED: Emergency department frailty index
HDU: High dependency unit
ICU: Intensive care unit
ISAR: Identification of seniors at risk
LOS: Length of stay
MEWS: Modified early warning score
NEWS: National early warning score
RCP: Royal college of physicians
SPSS: Statistical product and service solutions
TRST: Triage risk stratification tool
